# Efficacy and Safety of Praziquantel, Tribendimidine and Mebendazole in Patients with Co-infection of *Clonorchis sinensis* and Other Helminths

**DOI:** 10.1371/journal.pntd.0003046

**Published:** 2014-08-14

**Authors:** Li-Li Xu, Bin Jiang, Ji-Hui Duan, Shi-Feng Zhuang, Yong-Chun Liu, Shi-Qiao Zhu, Li-Ping Zhang, Hao-Bing Zhang, Shu-Hua Xiao, Xiao-Nong Zhou

**Affiliations:** 1 National Institute of Parasitic Diseases, Chinese Center for Disease Control and Prevention, Shanghai, People's Republic of China; 2 WHO Collaborative Center for Malaria, Schistosomiasis and Filariasis, Key Laboratory of Parasite and Vector Biology, Ministry of Health, Shanghai, People's Republic of China; 3 Hunan Center for Disease Control and Prevention, Changsha, People's Republic of China; 4 Qiyang Center for Disease Control and Prevention, Yongzhou, People's Republic of China; 5 Sichuan Center for Disease Control and Prevention, Chengdu, People's Republic of China; Case Western Reserve University School of Medicine, United States of America

## Abstract

**Background:**

Both tribendimidine and mebendazole are broad-spectrum drugs for anti-intestinal nematodes. We aim to assess the efficacy and safety of tribendimidine and mebendazole in patients with co-infection of *Clonorchis sinensis* and other helminths.

**Method:**

We performed a randomized open-label trial in Qiyang, People's Republic of China. Eligible participants were randomly assigned to one of four groups: (i) a single dose of 400 mg tribendimidine, (ii) 200 mg tribendimidine twice daily, (iii) 75 mg/kg praziquantel divided in four doses within 2 days, and (iv) a single dose of 400 mg mebendazole. Cure rates and egg reduction rates were assessed, and adverse events were monitored after treatments. Uncured patients accepted the second treatment with the same drugs after the first treatment.

**Results:**

156 patients were eligible for the study. Results from the first treatment showed that the cure rates of single-dose tribendimidine and praziquantel against *C. sinensis* were 50% and 56.8%, respectively; the single-dose tribendimidine achieved the cure rate of 77.8% in the treatment for hookworm, which was significantly higher than that of praziquantel; Low cure rates were obtained in the treatment of single-dose tribendimidine against *Ascaris lumbricoides* and *Trichuris trichiura* (28.6% and 23.1%). Results of the second treatment illustrated the cure rates of tribendimidine and praziquantel against *C. sinensis* were 78.1% and 75%, respectively. Most adverse events were mild and transient. Adverse events caused by tribendimidine were significantly less than praziquantel.

**Conclusion:**

Single-dose tribendimidine showed similar efficacy against *C. sinensis* as praziquantel with less adverse events, and achieved significantly higher cure rate in the treatment for hookworm than those of praziquantel and mebendazole. Low cure rates, which were still higher than other drugs, were obtained in the treatment of single-dose tribendimidine against *Ascaris lumbricoides* and *Trichuris trichiura*.

**Trial Registration:**

Controlled-Trials.com ISRCTN55086560

## Author Summary

Co-infection of *Clonorchis sinensis* and other helminths is common in places with poor settings. Preventive chemotherapy is commonly used to control the co-infection of helminths due to lack of effective vaccine. It is important to investigate the efficacy and safety of tribendimidine, a broad-spectrum anti-intestinal nematodes drug, against co-infection of *C. sinensis* and other helminths, in comparison with those of praziquantel and mebendazole. The cure rates of single-dose 400 mg tribendimidine against *C. sinensis*, hookworm, *Ascaris lumbricoides* and *Trichuris trichiura* in this study were 50%, 77.8%,28.6% and 23.1%, respectively. The single-dose tribendimidine achieved similar efficacy as the four-dose praziquantel in the treatment for *C. sinensis* with significantly less adverse events. Meanwhile, significantly higher cure rate of tribendimidine was found in the treatment for hookworm than other drugs. Most adverse events were mild and transient in this study. Tribendimidine seems a better drug choice for the patients co-infected with *C. sinensis* and other helminths than that of praziquantel.

## Introduction

Clonorchiasis is one of the neglected food-borne trematodiasis caused by infection of *Clonorchis sinensis (C. sinensis)*, which is mainly prevalent in East and Southeast Asia, especially in the People's Republic of China (P.R. China), the Republic of Korea, northern part of Vietnam, and the far eastern part of Russia [1]–[4]. An estimated 15 million people are globally infected with *C. sinensis*, more than 80% of whom (12.49 million) are Chinese [2], [5]–[8]. Because of social custom and unhealthy eating behaviors, more and more people were infected with *C. sinensis*, which significantly increased the burden of disease [9]–[11]. In some area, the prevalence of infection is even more than 65% [12]–[13]. Meanwhile, among those patients with *C. sinensis* infection, co-infection with other helminths, such as hookworm, *Ascaris lumbricoides (A. lumbricoides)* and *Trichuris trichiura (T. trichiura)*, is common in some low-income areas with poor sanitation. As a subgroup of neglected tropical diseases, these soil-transmitting helminths infections affect nearly 1.4 billion people worldwide [14]. Owing to the absence of effective vaccine, preventive chemotherapy is commonly used to control the co-infection of helminths and reduce the morbidity.

Praziquantel exhibits satisfactory efficacy and becomes the first line drug for clonorchiasis. The recommended treatment regimen by WHO is 25 mg/kg thrice daily for two consecutive days [15], which can achieve the cure rates of 93.5–100% [16]–[17]. However, this treatment regimen is difficult to complete in the mass treatment because of multiple treatments and adverse events [18], whereas administration of single dose or reduction of treatment course results in less or unstable efficacy [19]–[21]. In addition, praziquantel also exhibits activity against hookworm [22]. A cure rate of 93% was reported when a single dose of 40 mg/kg praziquantel was administrated to patients with hookworm infection [23]. Imidazole drugs are recommended for treatment of soil-transmitting helminths by WHO. Among these, mebendazole is a broad-spectrum anthelmintic agent. Mebendazole was reported to be effective against *C. sinensis* in rats by a single dose of 150 mg/kg as the complete curative dose [24]. Similar results were also reported in Xiao's study [25]. Tribendimidine is an abroad-spectrum anti-intestinal nematodes drug that recently appeared in Chinese market. In updated reports, tribendimidine is proved to be effective to *C. sinensis* in rats and hamsters. Mean worm burden reductions of the single dose of 150 and 100 mg/kg tribendimidine in the rats and in hamsters were 98% and 100%, respectively [26]. Meanwhile, tribendimidine showed effective activity to juvenile *C. sinensis* in hamsters, 90.6% of the mean worm burden reduction were achieved by using the dose of 100 mg/kg tribendimidine [25]–[29]. In addition to these laboratory studies, tribendimidine also showed good therapeutic profiles against *C. sinensis* and *Opisthorchis viverrini* in clinical trials, and only mild and transient adverse events were reported [30]–[31]. Based on the aforementioned evidences, we aim to assess the efficacy and safety of praziquantel, tribendimidine and mebendazole in patients with co-infection of *C. sinensis* with other helminths in this randomized open-label trial.

## Materials and Methods

### Ethical statement

The study was approved by the ethical review committee of the National Institute of Parasitic Diseases, Chinese Center for Diseases Control and Prevention (No. 201205). The trial was registered with Current Controlled Trials (ISRCTN55086560). Written informed consent was obtained from every participant or their guardian. We explained risk and benefits on the consent form. Participants were voluntary, and individuals could withdraw from the trial at any time.

### Study area and population

The study was conducted in the Qiyang County, Hunan province, P.R. China, from June to September in 2012. A total of 867 habitants aged 15 to 65 in Dazhongqiao village and Sankoutang village were enrolled in the preliminary survey.

### Eligibility criteria

All residents of Dazhongqiao village and Sankoutang village aged 15 to 65 years old were invited to provide one stool sample to perform the Kato-Katz thick smears. Common intestinal worm eggs including *C. sinensis*, hookworm, *A. lumbricoides* and *T. trichiura* were checked and counted under the light microscopy. Eligible for inclusion were those who were infected with more than one species of helminth and provided the written informed consent in preliminary survey, and then submitted the second stool sample before the treatment.

Participants could be excluded from treatment if fulfilling any of the following exclusion criteria: who was pregnant (of the females), present of any abnormal medical disorder (i.e., fever and hepatomegaly), historical record of any acute or sever chronic disease, was psychiatric and neurological disorders, and gave anthelmintic treatment within the previous 4 weeks.

### Sample size

Sample size was based on a suggested sample size of 12 patients per group for proof-of-concept trials [32]. Taking into account of dropping out, we planned to enroll 40 participants per group.

### Randomization and drugs

Participants who met all study criteria were randomly assigned to one of the four treatment groups by a computer-generated randomization number. The random number sequence was generated with SAS software (version 9.1) according to the list of identification number of 156 patients. Participants and trial designer were not masked to treatment allocation, but the laboratory teams were masked throughout the study.

Tribendimidine (200 mg tablets) was purchased from Shandong Xinhua Pharmaceutical Corporation (Zibo, Shandong, P.R. China); praziquantel (200 mg tablets) was donated by Nanjing Pharmaceutical Corporation (Nanjing, Jiangsu, P.R.China); mebendazole (100 mg tablets) was the product of Guangxi Yingkang Pharmacy CO., LTD (Nanning, Guangxi, P.R.China).

### Procedures

First, a total of 867 residents were invited to participate in the preliminary survey within ten days. The individual information including name, age, sex, educational background, race and telephone were recorded. Participants received containers with unique identification numbers and were invited to bring a fresh stool sample in the following morning. Patients with a microscopically confirmed co-infection with helminths were asked for a second stool sample. Eligible participants were examined by clinicians before drug administration, and women aged 15–49 years old accepted urine samples test to exclude the pregnancy.

Second, the first treatment was given to all eligible participants. Drugs were swallowed with clean water and accompanied by a small food item to improve tolerability and increase bioavailability. Praziquantel was administrated orally according to regional policy in Hunan province: 75 mg/kg in four divided doses (twice daily spaced by 6 h for 2 days). Tribendimidine was given by two means: one is a single dose of 400 mg and the other is 200 mg twice daily spaced by 6 h. Mebendazole was given 400 mg as a single dose. In addition, parts of participants who were treated with tribendimidine accepted the tests for blood and urine samples (including blood and urine common biochemical indexes, hepatic and renal function indexes) and ECG examinations before and 24 h after the treatment. Third, the adverse events (AEs) were monitored and recorded.

Participants were asked to report any potential drug-related signs and symptoms at 3 h, 24 h and 48 h after the first administration. Solicited adverse events, including headache, vertigo, vomiting, nausea, asthenia, dizziness, anxiety, allergic reactions, abdominal pain and fever, were recorded. Intensity of AEs were recorded and graded as mild, moderate, severe and serious as judged by clinicians. Three weeks after treatment, participants were asked for two consecutive stool samples.

Finally, the second treatment was given to participants who were still *C. sinensis* egg-positive after the first treatment with the same doses of praziquantel and tribendimidine within 6 weeks after the first administration due to ethical reason. We adopted the same clinical trial practice process as the first treatment including performance of written informed consent. A total of 52 participants accepted the retreatment. At last, worm egg-positive participants who were enrolled in our study were treated with corresponding drugs.

### Laboratory examinations

Filled stool containers were taken to the laboratory at the Qiyang Center of Diseases Control and Prevention (Qiyang CDC). From each stool sample, three Kato-Katz thick smears were prepared and were quantitatively examined with light microscopy for worm eggs. Numbers of worm eggs were counted and recorded for each parasite species separately. 5% of slides were re-examined randomly for quality control by a senior microscope technician.

Blood and urine samples collected from some participants were taken to the local hospital with ice packs. Urine samples and parts of blood samples were tested within 30 min to examine the changes of common biochemical indexes. The other blood samples were stored at 4°C in the refrigerator for 4 h, and then were centrifugalized. The supernatants were tested for hepatic and renal function detection.

### Primary and secondary outcomes

Primary outcomes were the cure rates (CRs) and egg reduction rates (ERRs) at 3 weeks after treatments as efficacy outcomes. The CR was defined as the percentage of participants excreting eggs before treatment who became negative after treatment. The ERR was defined as the group's reduction of geometric mean egg count after treatment divided by the geometric mean of the same patients before treatment, multiplied by 100. Secondary outcomes were the frequencies of AEs and the pathological changes of results of biochemical tests and ECG examination after treatments.

### Statistical analysis

All data were double entered, and the per-protocol analysis was pursued. Statistical analyses were performed with SAS software (version 9.1, Statistical Analysis System, RTI, Cary, North Carolina, USA). The numbers of each kind of worm eggs recorded from 6 Kato-Katz slides before and after treatment were added to calculate the arithmetic mean of eggs per gram of stool (EPG) for every participant. The arithmetic means was used to determine the infection intensity, and the geometric EPG was calculated to assess the egg reduction rate among the treatment groups. Prevalence of *C. sinensis* was stratified, according to the classification of infection intensities, into three catalogues, e.g. light (1–999 EPG), moderate (1000–9999 EPG), and severe (>10000 EPG) infections [33]. Prevalence of hookworm was stratified into three catalogues, e.g. light (1–1999 EPG), moderate (2000–3999 EPG), and severe (>4000 EPG) infections, in accordance of the classification put forth by WHO [15]. Logistic regression model was used to examine cure rates of helminths in different treatment groups. Pearson's χ^2^ test was applied to compare the proportion of reported adverse events between the treatment groups. Negative binomial regression models were used to compare the numbers of adverse events in the treatment groups.

## Results

### Study cohort

**Figure 1:**
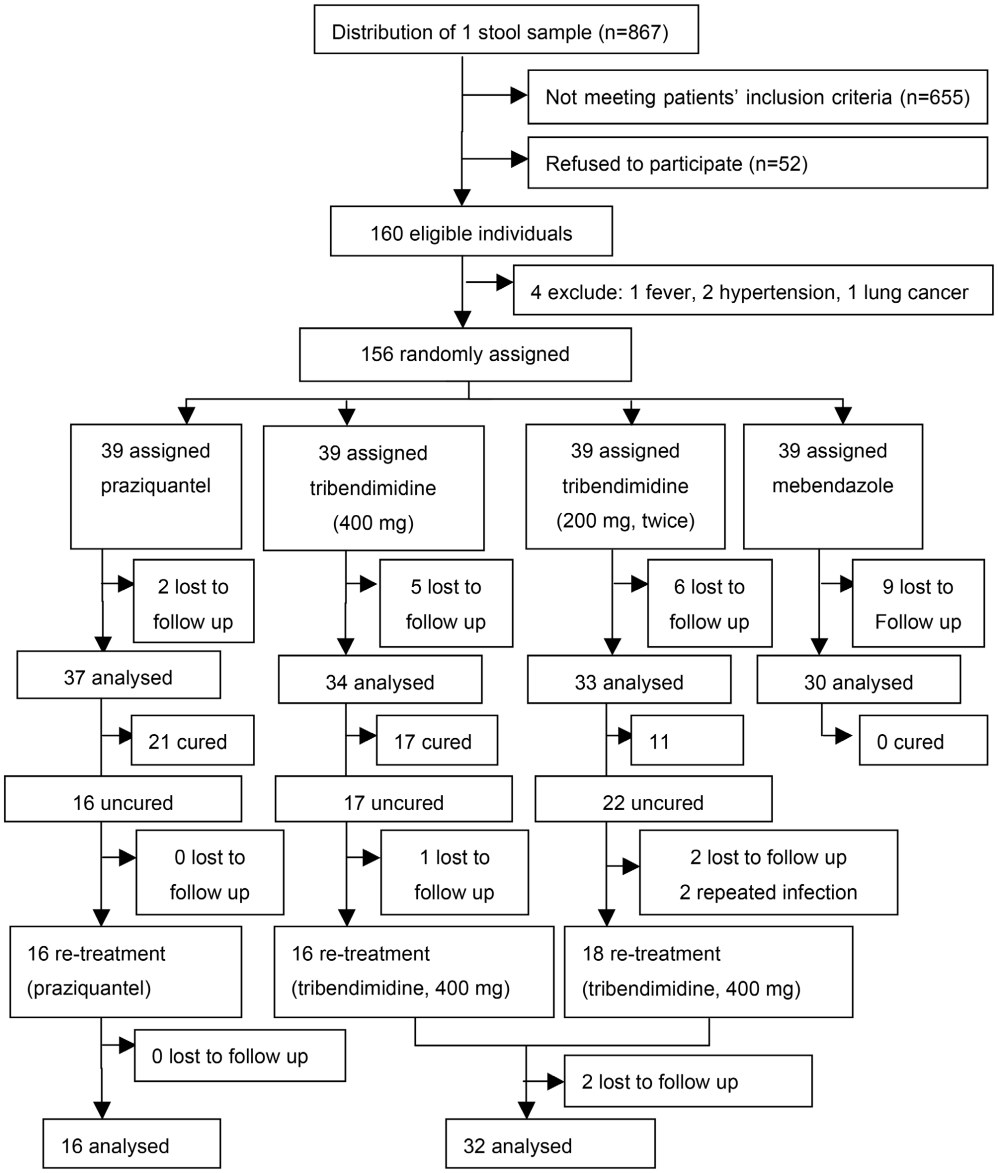
Flow diagram of the randomized controlled trial.

Among 160 patients invited for the treatment, 4 were excluded (Figure 1), because one had fever, two had hypertension and the other had lung cancer. Thus, a total of 156 patients were randomly assigned to 1 of 4 treatment arms, among them 22 patients (14.1%) were lost to follow up. Among 55 uncured patients after the first treatment, 3 were lost to follow up and 2 were identified as repeated infections. Then 50 persons accepted the second treatment. After the treatment, 2 patients dropped out for the study, and the complete data records for the final analysis were 48.

### Preliminary survey

All baseline characteristics of treatment groups were similar in the first treatment. The mean age of the 156 patients was 53.5 years (Table 1). All the participants were infected with *C. sinensis*, and the intensity of *C. sinensis* infections was mainly moderate. The *C. sinensis* geometric mean egg counts ranged from 120 to 15779.7 EPG. The proportion of patients co-infected with hookworm ranged from 61.5% to 71.8%, and most of patients were lightly infected. The hookworm geometric egg counts ranged from 24 to 2839.8 EPG. The proportion of concurrent infections with *A. lumbricoides* and *T. trichiura* were between 30.8% and 41.0% with mild intensity of infection.

**Table 1:** Demographic and laboratory baseline characteristics of 156 patients with co-infection of *C. sinensis* and other helminths.

	Praziquantel (n = 39)	Tribendimidine400 mg (n = 39)	Tribendimidine 200 mg (n = 39)	Mebendazole (n = 39)
**Characteristics**				
**Male**	19	17	16	22
**Female**	20	22	23	17
**Mean age**	56.1	54.9	52.4	50.5
**(SD) age, years**	(7.6)	(8.8)	(8.5)	(11.5)
***C. sinensis*** ** infection**				
**Number of people infected**	39	39	39	39
**Overall GM eggs per g of stool**	2056.5	2100.3	2363.6	2051.4
**GM eggs per g of stool (range)**	120–12054.5	207.1–15779.7	351.5–10874.3	291.5–12938.3
**Number of people with light infection** *	9 (23.1%)	7 (17.9%)	6 (15.4%)	9 (23.1%)
**Number of people with moderate infection**	22 (56.4%)	24 (61.5%)	28 (71.8%)	24 (61.5%)
**Number of people with heavy infection**	8 (20.5%)	8 (20.5%)	5 (12.8%)	6 (15.4%)
**Co-infection with Hookworm infection**				
**Number of people infected**	24 (61.5%)	27 (69.2%)	28 (71.8%)	25 (64.1%)
**Overall GM eggs per g of stool**	173.5	241.2	220.4	219.7
**GM eggs per g of stool (range)**	24–2035.0	43.6–2839.8	24–2647.1	24–2054.9
**Number of people with Light infection** #	23 (95.8%)	25 (92.6%)	28 (90.3%)	24 (96%)
**Number of people with Moderate infection**	1 (4.2%)	2 (7.4%)	3 (9.7%)	1 (4%)
**Co-infection with ** ***Ascaris lumbricoides***				
**Number of people infected**	12 (30.8%)	16 (41.0%)	15 (38.5%)	14 (35.9%)
**Overall GM eggs per g of stool**	370.4	314.3	412.3	358.2
**GM eggs per g of stool (range)**	59.2–2184.1	51.7–1659.4	85.4–1476.0	72–1306.7
**Co-infection with ** ***Trichuris trichiura***				
**Number of people infected**	13 (33.3%)	14 (35.9%)	14 (35.9%)	15 (38.5%)
**Overall GM eggs per g of stool**	168.0	206.9	192.5	227.8
**GM eggs per g of stool (range)**	41.0–683.9	30.2–798.1	24–571.8	69.2–428.2

Data are number (%) of patients. GM = geometric mean.

*and # According to guideline's classification put forward by WHO, based on Kato-Katz analysis.

The two groups of treatment with praziquantel and tribendimidine were not equal in size in the second treatment. 16 patients accepted praziquantel treatment while 34 patients in the tribendimidine group. The mean age was 50.9 years (Table 2), and the intensity of infection in each group was mild. The geometric mean egg counts of *C. sinensis* ranged from 4 to 216.1 EPG. Prevalence of co-infection with hookworm ranged from 58.8% to 75%.

**Table 2:** Laboratory baseline characteristics of patients infected with *C. sinensis* in the second treatment.

	Praziquantel (n = 16)	Tribendimidine (n = 34)
**Characteristics**		
Male	7	15
Female	9	19
Mean year (SD age)	52.2(6.6)	50.3(7.1)
***C. sinensis*** ** infection**		
Overall GM eggs per g of stool	36.8	45.3
GM eggs per g of stool (range)	(4–202.2)	(8–216.1)
**Co-infection with hookworm**		
Number of people infected	12(75%)	20(58.8%)

### Cure rate (CR) and egg reduction rate (ERR) in the first treatment

First, the CRs of four groups with three drugs against *C. sinensis* were observed. 56.8% of CR were obtained in the praziquantel group (Table 3), followed by the single-dose tribendimidine (50%) and 200 mg tribendimidine twice daily (33.3%) which were significantly lower than that of praziquantel (OR = 0.38, 95% CI 0.14–1.01, P = 0.05) (Table 4). But no significant difference was observed between praziquantel and single-dose tribendimidine (P>0.05). The ERRs of these groups were similar, with 98.0% of ERRs for praziquantel treatment, 98.3% of the single-dose tribendimidine, and 97.1% of tribendimidine 200 mg twice. No patients were cured in mebendazole group. Meanwhile, the CRs of these drugs administrated to patients with mild *C. sinensis* intensity were significantly higher than that with heavy infection (P = 0.024 and P = 0.045).

**Table 3:** Per-protocol analysis of prevalence and cure rates of praziquantel, tribendimidine and mebendazole in patients co-infected with *C. sinensis* and other helminths at follow-up, with Kato-Katz smear technique.

	Praziquantel (n = 37)	Tribendimidine400 mg (n = 34)	Tribendimidine200 mg twice (n = 33)	Mebendazole (n = 30)
***C. sinensis***				
**Patients cured**	21 (56.8%)	17 (50%)	11 (33.3%)	0 (0)
**GM egg per g of Stool**	41.3	36.1	69.4	591.2
**Egg reduction rate**	98.0%	98.3%	97.1%	71.2%
**Co-infection with hookworm**	n = 22	n = 22	n = 23	n = 18
**Patients cured**	4 (18.2%)	14 (63.6%)	11 (47.8%)	0 (0)
**GM egg per g of Stool**	116.6	53.7	76.3	77.8
**Egg reduction rate**	32.8%	77.8%	65.4%	64.6%
**Co-infection with ** ***A. lumbricoides***	n = 12	n = 14	n = 12	n = 13
**Patients cured**	2 (16.7%)	4 (28.6%)	0 (0)	1 (7.7%)
**GM egg per g of Stool**	122.7	90.9	110.9	73.5
**Egg reduction rate**	66.9%	71.1%	73.1%	79.5%
**Co-infection with ** ***T. trichiura***	n = 13	n = 13	n = 12	n = 14
**Patients cured**	0 (0)	3 (23.1%)	4 (33.3%)	0 (0)
**GM egg per g of Stool**	78.8	45.8	38.4	82.5
**Egg reduction rate**	53.1%	77.9%	80.1%	63.8%

**Table 4:** Logistic regression analysis of CRs between praziquantel and tribendimidine groups in first treatment.

Per-protocol CR analysis	OR (95%CI)	*p*
***C. sinensis*** ** infection**		
**Tribendimidine (400 mg) vs Praziquantel**	0.76 (0.30–1.94)	0.57
**Tribendimidine (200 mg, twice) vs Praziquantel**	0.38 (0.14–1.01)	0.05
**Tribendimidine (400 mg) vs Tribendimidine (200 mg, twice)**	2 (0.74–5.37)	0.17
**Co-infection with hookworm**		
**Tribendimidine (400 mg) vs Praziquantel**	7.88 (1.96–31.57)	0.004
**Tribendimidine (200 mg, twice) vs Praziquantel**	4.09 (1.03–16.28)	0.046
**Tribendimidine (400 mg) vs Tribendimidine (200 mg, twice)**	0.52 (0.15–1.76)	0.293

Abbreviations: CI, confidence interval.

Second, CRs of the single dose and 200 mg twice daily of tribendimidine against hookworm were 63.6% and 47.8%, respectively (Table 3). Two doses of tribendimidine achieved significantly higher CRs than that of praziquantel in the treatment for hookworm (CR 18.2%, OR = 7.87, 95% CI 1.96–31.57, P = 0.01, and OR = 4.09, 95% CI 1.03–16.28, p = 0.046) (Table 4). However, no significant difference was found between two tribendimidine treatment groups. No patient was cured in mebendazole group. The highest ERR was achieved in the single dose of tribendimidine (77.8%), followed by tribendimidine 200 mg twice daily (65.4%), mebendazole (64.6%) and praziquantel (32.8%).

Third, low CRs were found in the treatments against *A. lumbricoides* by praziquantel, the single dose of tribendimidine, and mebendazole, of which CRs were 16.7%, 28.6% and 7.7%, respectively (Table 3). No significant difference was found among these groups. No patients were cured in tribendimidine 200 mg twice daily. Four treatment groups achieved moderate ERRs ranged from 66.9% to 79.5%, and no statistically difference was found among these treatment groups.

Fourth, the CRs of the single dose and 200 mg twice daily of tribendimidine against *T. trichiura* were 23.1% and 33.3%, respectively. The respective ERRs were 77.9% and 80.1% (Table 3). No patients were cured in praziquantel and mebendazole groups, but respective ERRs were 53.1% and 63.8%. No statistically significant difference was observed in the comparison of CRs or ERRs among four groups.

**Figure 2:**
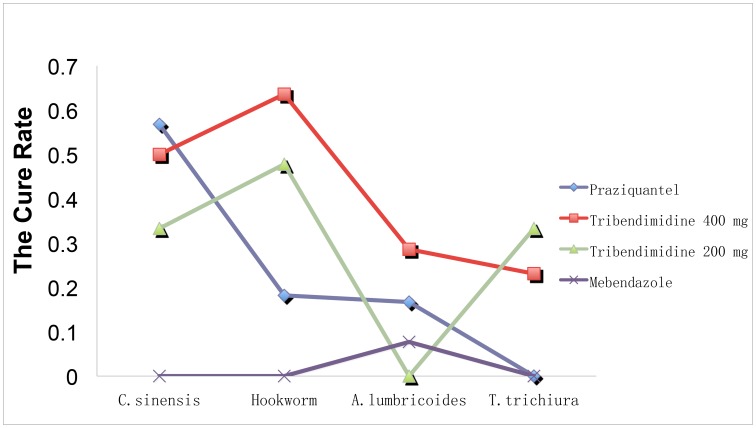
The cure rates (CRs) of praziquantel, tribendimidine and mebendazole against *C. sinensis*, hookworm, *A. lumbricoides* and *T. trichiura*.

In total, tribendimidine achieved higher CRs against hookworm, *A. lumbricoides* and *T. trichiura* in comparison with that of other drugs (Figure 2), and a similar CR against *C. sinensis* as that of praziquantel.

### CR and ERR in the second treatment

First, high CRs against *C. sinensis* were achieved in praziquantel (75%) and tribendimidine (78.1%) groups (Table 5), with similar ERRs between respective two groups (75.8% and 74.2%). Second, the CRs of praziquantel and tribendimidine against hookworm were 16.7% and 55%, respectively. There is significantly difference between two groups (OR = 5.4, 95% CI 0.98–29.91, P = 0.05)(Table 6).

**Table 5:** Per-protocol analysis of prevalence and CRs of praziquantel and tribendimidine in patients infected with *C. sinensis* at follow-up in the second treatment.

	Praziquantel	Tribendimidine
***C. sinensis*** ** infection**		
Patients cured/patients infected	12/16(75%)	25/32(78.1%)
GM eggs per g of stool	8.9	11.7
GM eggs per g of stool (range)	(4–16)	(4–28)
Egg reduction rate	75.8%	74.2%
**Co-infection with Hookworm**		
Patients cured/patients infected	2/12(16.7%)	11/20(55%)

**Table 6:** Logistic regression analysis of CR between praziquantel and tribendimidine groups in second treatment.

Per-protocol analysis	*C. sinensis* infection		Co-infection with hookworm	
	OR (95%CI)	*p*	OR (95%CI)	*p*
Tribendimidine vs Praziquantel	1.19 (0.29–4.87)	0.81	5.4 (0.98–29.91)	0.05

### Adverse events

Adverse events were assessed at 3 h, 24 h and 48 h after each treatment. No symptom was reported before treatment. Most of AEs were mild and transient. In total, 45 (43.3%) mild, 3 (2.9%) moderate and 2 (1.9%) severe AEs were reported in the first treatment (Table 7), and 17 (35.4%) mild and2 (4.2%) moderate AEs were found in the second treatment. AEs of two tribendimidine groups were significantly less than that of praziquantel (p = 0.034 and p = 0.0002) (Table 8).

**Table 7:** Summary of clinical symptoms recorded after drug administration, stratified by treatment groups.

Adverse Events Grade	Mild	Moderate	Severe	Serious
**First treatment**				
**Praziquantel**	24 (64.9%)	2 (5.4%)	1 (2.7%)	0
**Tribendimidine 400 mg**	6 (17.6%)	1 (2.9%)	1 (2.9%)	0
**Tribendimidine 200 mg,twice**	4 (12.1%)	0	0	0
**Total**	45 (43.3%)	3 (2.9%)	2 (1.9%)	0
**Second treatment**				
**Praziquantel**	8 (50%)	1 (6.3%)	0	0
**Tribendimidine**	9 (28%)	1 (2.8%)	0	0
**Total**	17 (35.4%)	2 (4.2%)	0	0

**Table 8:** Negative binomial regression analysis of adverse events recorded within 48 h after drug administration in the first treatment.

Parameter	Estimate	Standard error	95%CI	*p*
First treatment				
Group 1	−0.9515	0.3248	−1.5881–−0.3149	0.034
Group 2	−1.9333	0.5141	−2.9409–−0.9257	0.0002

Group 1: the single-dose tribendimidine vs praziquantel;

Group 2: tribendimidine 200 mg twice daily vs praziquantel.

Most of reported AEs in the tribendimidine group were vertigo, headache, nausea, fatigue and anxiety. Severe vomiting and drug allergy events were found in the praziquantel and the single dose of tribendimidine groups, respectively. Among these AEs, vertigo was more common in the praziquantel group (35.1%), which was significant higher than that of tribendimidine (p = 0.03). Patients who had AEs were treated with antiemetics and an antiallergic agent to reduce the symptoms.

### Biochemical and ECG examinations

A total of 18 patients in the single dose of tribendimidine group and 20 patients treated with the other dose of tribendimidine accepted tests of the blood and urine samples, and ECG examination before and 24 h after treatment. No pathological changes were found from those results of biochemical and ECG examinations after tribendimidine treatments.

## Discussion

The efficacy outcomes of our study demonstrate that tribendimidine is as efficacious as praziquantel for treatment of *C. sinensis* infection. Similar results have been reported in the treatments of *C. sinensis* and *O. viverrini* with tribendimidine and praziquantel [30]–[31]. Meanwhile, higher CR can be achieved when patient who has mild intensity of *C. sinensis* infection were treated with tribendimidine in the first and the second treatments. High ERRs were obtained in the first treatment, which means tribendimidine can reduce the intensity of infection although can not eliminate the infection for those uncured patients. Taking into account of higher CR obtained in patients with mild infection, we believe that increasing the number of treatment time can enhance the CR of tribendimidine against *C. sinensis*. In addition, a significant higher CR was obtained in tribendimidine against hookworm compared to those of praziquantel and mebendazole. As to *A. lumbricoides* and *T. trichiura*, more than 70% of ERRs were achieved in single-dose tribendimidine group. Despite of low efficacy, the CRs of tribendimidine were still higher than those of praziquantel and mebendazole. Based on the above results, tribendimidine seems to show relative better efficacy against co-infection of helminths than that of praziquantel and mebendazole. Tribendimidine, first discovered and invented in China, is an amidantel derivative and has a broad spectrum of activity against infections of intestinal nematodes, e.g., hookworm and *A. lumbricoides* [29]. Tribendimidine has been proved to be an L-subtype nicotinic acetylcholine receptor agonist, similar to levamisole and pyrantel [34]. The p- (1-dimethylamino ethylimino) aniline and acetylated deacylated amidantel, as the metabolites of tribendimidine, are completely broken down and eliminated within 24 h, and no original compound of tribendimidine was found in plasma, urine, and feces of healthy volunteers administered orally with tribendimidine. The maximal plasma concentration after administration of 200 mg and 400 mg tribendimidine in healthy Chinese were 0.37 and 0.64 mg/L, and the half-life period was about 4 to 5 h [35]–[36]. The concentration of 0.1 ug/mL tribendimidine can kill adult worm in the vitro effect of tribendimidine against *C. sinensis* infection [37]. Therefore, the maximal plasma concentration after administration of 200 mg tribendimidine is more than the minimal concentration of tribendimidine to kill *C. sinensis* in the vitro. According to these facts, we designed two different doses of tribendimidine in the study. However, we did not get the satisfactory results since only 33.3% of CR was obtained when we used the dose of 200 mg twice daily to treat patients infected with *C. sinensis*. This reason may be that adult worms parasitize in the bile duct, and drug concentration in the bile is lower than that in the plasma.

As mentioned before, praziquantel is the first choice for *C. sinensis* and showed the activity against hookworm in some reports. For instance, about 80%–95% of CRs were reported in the treatment of praziquantel against *C. sinensis* infections [20], [38]. However, only 56.8% of CR was found in our trial, it is because following reasons. First, we used less doses of praziquantel than that recommended by WHO. This dose-choosing is based on the same treatment regimen as that used in the study area. Second, the higher intensity of *C. sinensis* infections for those patients who received treatment with praziquantel may be another reason. In order to compare the efficacy with same dose of tribendimidine, we also adopted the same single dose of 400 mg mebendazole instead of recommended dose by WHO (a single dose of 500 mg), no patients were cured in mebendazole groups. Taking into account of low absorption characteristic of mebendazole, reducing dose resulted in reduced efficacy.

Results from our study on AEs, both tribendimidine and praziquantel revealed to be well tolerated at the dosage of the trial, and most of AEs observed were mild and transient. However, the numbers of AEs caused by tribendimidine were significantly less than that of praziquantel. Patients treated with tribendimidine were less likely to experience vertigo than that with praziquantel treatment. No pathological changes were found in patients who accepted tribendimidine treatment. These outcomes illustrated tribendimidine is a safe drug for human use at the dosage of the trail. However, apart from the frequent reported adverse events, such as adverse reactions of nervous system and gastrointestinal system, caused by tribendimidine [39]–[41], we only observed a severe drug allergy reaction. Allergy symptoms appeared in 18 h after single-dose 400 mg tribendimidine treatment and disappeared in 7 days after its emergency.

In conclusion, one single dose of 400 mg tribendimidine shows similar therapeutic profiles as praziquantel against *C. sinensis* in this trial. It is benefit for preventive chemotherapy of *C. sinensis* infections in places with high prevalence. However, large-scale clinical study is warrant to perform in order to further verify the efficacy and appraise the safety. Meanwhile, taking into account of good efficacy of tribendimidine against hookworm, it has particularly noticed that tribendimidine is a better choice to cure patients with co-infection of *C. sinensis* and hookworm.

## Supporting Information

Text S1 The trial protocol.
(DOC)

Text S2 CONSORT checklist.
(DOC)
