# Supplementary material for: Efficacy and Safety of Praziquantel, Tribendimidine and Mebendazole in Patients with Co-infection of Clonorchis sinensis and Other Helminths
Source: PLoS Negl Trop Dis. 2014 Aug 14;8(8):e3046. doi: 10.1371/journal.pntd.0003046 (PMC4133228; doi:10.1371/journal.pntd.0003046)
Supplement: Text S1 — The trial protocol. (DOC) [file pntd.0003046.s001.doc]

**The Protocol**

***1. The objective***

To appraise the efficacy and safety of praziquantel, tribendimidine and mebendazole against the co-infection of helmithes.

***2. The background***

Co-infection with food-borne helminthes (such as *Clonorchis sinensis* , hookworm, *Ascaris lumbricoides* and *Trichuris trichiura*) is common in China. As a subgroup of neglected tropical diseases, these soil-transmitting helminthes infections affect nearly 1.4 billion people worldwide. Among these, approximately 129 million patients are Chinese. Owing to the absence of a vaccine, chemotherapy is commonly used to control the coinfection of helminthes. Thus, the selection of drugs is crucial. As to C. sinensis, praziquantel exhibits satisfactory efficacy and becomes the first line drug of choice for clonorchiasis. The recommended treatment regimen by WHO is 25 mg/kg thrice daily for two consecutive days that results in cure rates of 93.5–100%. However, this dose schedule of praziquantel may be difficult to complete in the mass treatment because of multiple treatments and adverse events, while administration of single dose or reduction of treatment course results in less or unstable efficacy. What’s more, although no praziquantel resistance cases are documented, a low cure rate (29%) reported in Vietnam as patients with C. sinensis infection were treated with three daily 25 mg/kg of praziquantel prompted us to pay more attention to the resistance to praziquantel. Therefore, developing the new drugs against C. sinensis is urgent. In addition, praziquantel also exhibits activity against hookworm and 93% cure rate was reported as a single dose of 40mg/kg praziquantel was administrated to patients with hookworm infection. As to soil-transmitting helminthes, imidazole drugs are recommended for treatment by WHO. Among these, mebendazole is a broad-spectrum antihelmintic agent and was reported to be effective against C. sinensis in rats and their single complete curative dose was 150 mg/kg. Similar results were also reported in Xiao’s study. Tribendimidine is abroad-spectrum anti-intestinal nematodes drug. In recent researches, it is proved to be effective to C.sinensis in rats and hamsters. A single dose of 150 and 100 mg/kg tribendimidine resulted in 98% and 100% mean worm burden reduction in the rats and in hamster, respectively. Meanwhile, tribendimidine showed good effective activity to juvenile C. sinensis in hamsters, and the dose of 100mg/kg tribendimidine caused 90.6% mean worm burden reduction. In addition to these laboratory studies, tribendimidine also showed good therapeutic profiles against C. sinensis and Opisthorchis viverrini in clinical trials, and only mild and transient adverse events were reported. Based on the above evidence, we use the praziquantel, tribendimidine and mebendazole to treat the patients with co-infection of helminthes to appraise the efficacy and safety.

***3. The ethical application***

To apply for approval of ethical verification from the ethical review committee of the National Institute of Parasitic Diseases, Chinese Center for Diseases Control and Prevention with the related supporting information.

***4. The registration of clinical trial***

To register this clinical trial on the website of [www.controlled-trials.com](http://www.controlled-trials.com/)

***5. The procedure***

**(1) The study design**: we will perform a randomized, open-label trial to assess the efficacy and safety of praziquantel, tribendimidine and mebendazole against the co-infection of helmithes. All the participants who meet the eligible criteria will be randomly assigned into four groups: Praziquantel will be administrated orally according to regional policy in Hunan: 75 mg/kg in four divided doses (twice daily spaced by 6 h for two consecutive days). Tribendimidine will be given by two means: one is a single dose of 400 mg and the other is 200 mg twice daily spaced by 6 h. Mebendazole will be given 400 mg as a single dose.

In addition, parts of participants who are assigned to be treated with tribendimidine will accept blood and urine routine checks, hepatic and renal function examinations, and electrocardiogram (ECG) examinations before and 24 h after the treatment. The abnormal indexes will be supervised and recorded.

Participants will be asked to report any potential drug-related signs and symptoms at 3 h, 24 h and 48 h after the first dose by the use of a standardized questionnaire.

Three weeks after treatment, participants were asked for two consecutive stool samples.

**(2) Preliminary survey:** To invite the residents aged 15 to 65 who lives in the Dazhongqiao and Sankoutang village , Qiyang country, Hunan province ,P.R.China to participate this trial. The name, age, sex, educational background, race and telephone will be recorded. Participants will receive containers with unique identification numbers and be invited to bring a fresh stool sample the following morning. Stool containers will then be taken to the laboratory at the Qiyang Center of Diseases Control and Prevention (Qiyang CDC). From each stool sample, three Kato-Katz thick smears will be prepared and be quantitatively examined with light microscopy for worm eggs (e.g., C. sinensis, hookworm, A. lumbricoides and T. trichiura). Number of worm eggs are counted and recorded for each parasite species separately. Patients with a microscopically confirmed co-infection with helminthes will be asked for a second stool sample.

**(3) Sample size.** Sample size was based on a suggested sample size of 12 patients per group for proof-of-concept trials. Taking into account of the patients dropping out, we will plan to enroll 40 participants per group.

**(4) Eligibility criteria**: Eligible for inclusion is all residents of Dazhongqiao village and Sankoutang village aged 15 to 65 years old whose first stool sample to perform the Kato-Katz thick smears in preliminary survey, co-infection with helminthes, and submit the second stool sample and the written informed consent before the treatment.

**(5) Exclusion criteria**: Participants will be excluded from treatment if fulfilling any of the following exclusion criteria: pregnant (for females), the presence of any abnormal medical disorder (i.e., fever and hepatomegaly), history of any acute or sever chronic disease, psychiatric and neurological disorders, and anthelmintic treatment within the previous 4 weeks.

**(6) Informed consent**: Each of the participants will receive a written informed consent. We explain the risk and benefits on the consent form, and tell them all participants are voluntary, and individuals can withdraw from the trial at any time. If the participant is a child, the informed consent should be signed by his/her legal guardian. Written informed consent need to be obtained from each participant or legal guardians of each child before they are allowed to participate this trial.

**(7) The check-up before treatment**: all participants will be required to undergo a inquisitorial medical check-up, and the female participants aged 16 to 50 will accept the pregnancy test. Participant assigned to the two tribendimidine treatment groups will accept blood and urine routine checks, hepatic and renal function examinations, and electrocardiogram (ECG) examinations before the treatment.

Blood samples should be taken immediately to the local hospital with ice packs. Urine samples and part of blood samples should be tested within 30 min as urine and blood routine checks. The other blood samples should be stored at 4 ^◦^C in the refrigerator for 4 h, and then be centrifugalized. The supernatants will be tested for hepatic and renal function detection.

**(8) Take the medication**: The investigator should give the medication to participants, and ensure they will swallow it with warm water. The participant cannot take any other drugs during the experiment. If the drug is spit out within one hour after taking the medication, he/she will need to take the medication again. Both the strenuous exercise and the drinking will be forbidden during the experiment.

**(9) Follow up:** The trial clinicians will be present, monitor acute adverse events, and provide medical assistance if necessary. Participants will be asked to report any potential drug-related signs and symptoms at 3 h, 24 h and 48 h after the first dose by the use of a standardized questionnaire. Intensity of AEs are recorded and graded as mild, moderate, severe and serious as judged by study physicians.

Mild: asymptomatic or mild symptoms; clinical or diagnostic observations only; intervention not indicated

Moderate: minimal, local or noninvasive intervention indicated.

Severe: severe or medically significant but not immediately life-threatening; hospitalization or prolongation of hospitalization indicated.

Serious: life-threatening consequences; urgent intervention indicated.

**(10) Re-examination of the stool sample**: Three weeks after treatment, participants were asked for two consecutive stool samples. Stool containers will then be taken to the laboratory at the Qiyang Center of Diseases Control and Prevention (Qiyang CDC). From each stool sample, three Kato-Katz thick smears will be prepared and be quantitatively examined with light microscopy for worm eggs (e.g., C. sinensis, hookworm, A. lumbricoides and T. trichiura). Number of worm eggs will be counted and recorded for each parasite species separately.

**(11) Primary and secondary outcomes:** Primary outcomes are the cure rate (CR) and egg reduction rate (ERR) at 3 weeks after treatment as efficacy outcomes. The CR is defined as the percentage of participants excreting eggs before treatment who became negative after treatment. The ERR is calculated as the reduction in the group’s geometric mean egg count after treatment divided by the geometric mean of the same patients before treatment, multiplied by 100. Secondary outcomes are the frequencies of adverse events (AEs) and the abnormal biochemical tests after treatment including urine and blood test, hepatic and renal function examinations and ECG.

**(12) Statistical analysis.** All data will be double entered, and the per-protocol analysis will be pursued. Statistical analyses will be done with SAS software (version 9.1). The numbers of every kind of worm eggs recorded from 6 Kato-Katz slides before and after treatment will be added to calculate the arithmetic mean of eggs per gram of stool (EPG) for every participant. The arithmetic means is used to determine the infection intensity, and the geometric EPG is calculated to assess the egg reduction rate among the treatment groups. Logistic regression models will be used to examine cure rates of helminthes in different treatment groups. Negative binomial regression analysis will be adopted to compare egg reduction rates of helminthes between four groups. Pearson’s χ^2^ test will be applied to compare the proportion of reported adverse events between the treatment groups. Negative binomial regression models will be used to compare the number of adverse events in the treatment groups.

***6. The second treatment***

Based on first treatment results, we may carry out the second treatment with the same procedure and same clinical practices, hoping to enhance the efficacy.

***7. Ethical treatment***

If participants who accepted two treatments still infected with helminth, we will give them corresponding drugs recommended by doctors.

***8. The quality of control***

All the study clinicians, laboratory team and clinical investigator will be informed the study objects, eligibility criteria, procedures, benefits, and potentials risks. Each investigator is responsible for 10 participants. Each form will need the signatures of both the investigator and the one who will check them. During the whole trial, there will be some inspectors to visit the sites to ensure that all the contents of the trial will be strictly adhered to, and the original data will be checked too. Five percent of slides will be re-examined randomly for quality control by a senior staff.

***9. Preparation for the AEs***

In order to deal with the possible cases of severe adverse reaction caused by the treatment, cooperative hospital should prepare an ambulance. When it is necessary, the ambulance will immediately rush to the scene. Some drugs will be prepared, such as epinephrine injection, antihistamine, antiemetic, antipyretic analgesic, antispasmodic drugs and other conventional aid medicines.
